# Signatures of selection in natural populations adapted to chronic pollution

**DOI:** 10.1186/1471-2148-8-282

**Published:** 2008-10-10

**Authors:** Larissa M Williams, Marjorie F Oleksiak

**Affiliations:** 1 Department of Environmental and Molecular Toxicology, North Carolina State University, Box 7633, Raleigh, NC 27695-7633, USA; 2 Division of Marine Biology and Fisheries, Rosenstiel School of Marine and Atmospheric Sciences, University of Miami, 4600 Rickenbacker Causeway, Miami, FL 33149, USA

## Abstract

**Background:**

Populations of the teleost fish *Fundulus heteroclitus *appear to flourish in heavily polluted and geographically separated Superfund sites. Populations from three Superfund sites (New Bedford Harbor, MA, Newark Bay, NJ, and Elizabeth River, VA) have independently evolved adaptive resistance to chemical pollutants. In these polluted populations, natural selection likely has altered allele frequencies of loci that affect fitness or that are linked to these loci. The aim of this study was to identify loci that exhibit non-neutral behavior in the *F. heteroclitus *genome in polluted populations *versus *clean reference populations.

**Results:**

To detect signatures of natural selection and thus identify genetic bases for adaptation to anthropogenic stressors, we examined allele frequencies for many hundreds of amplified fragment length polymorphism markers among populations of *F. heteroclitus*. Specifically, we contrasted populations from three Superfund sites (New Bedford Harbor, MA, Newark Bay, NJ, and Elizabeth River, VA) to clean reference populations flanking the polluted sites. When empirical F_ST _values were compared to a simulated distribution of F_ST _values, 24 distinct outlier loci were identified among pairwise comparisons of pollutant impacted *F. heteroclitus *populations and both surrounding reference populations. Upon removal of all outlier loci, there was a strong correlation (R^2 ^= 0.79, p < 0.0001) between genetic and geographical distance. This apparently neutral evolutionary pattern was not evident when outlier loci were included (R^2 ^= 0.092, p = 0.0721). Two outlier loci were shared between New Bedford Harbor and Elizabeth River populations, and two different loci were shared between Newark Bay and Elizabeth River populations.

**Conclusion:**

In total, 1% to 6% of loci are implicated as being under selection or linked to areas of the genome under selection in three *F. heteroclitus *populations that reside in polluted estuaries. Shared loci among polluted sites indicate that selection may be acting on multiple loci involved in adaptation, and loci shared between polluted sites potentially are involved in a generalized adaptive response.

## Background

The genetic basis of adaptation is a fundamental issue in evolutionary biology. Much of the research in this field has been focused on the classic model systems of *Drosophila *[1-13] and *Arabidopsis *[14-18]. Recently, insight into adaptation in non-model species has become possible due to advances in molecular biology and statistics [19-31]. This recent expansion into studies of non-model systems allows further development of evolutionary inferences [32], such as the role that selection, mutation, gene flow, and drift play in adaptation [33]. A powerful approach to understand genome-wide adaptation is to investigate independent natural populations that inhabit environments with strong selective pressures.

One species that has adapted to a wide range of estuarine environments is the teleost fish, *Fundulus heteroclitus *[34]. *F. heteroclitus *is widely distributed along the United States' eastern seaboard from the Gulf of St. Lawrence to northeastern Florida [35]. Subpopulations of *F. heteroclitus *inhabit clean estuaries as well as those heavily impacted by chemical pollutants (reviewed in [36]). Three well-known polluted sites where *F. heteroclitus *reside are New Bedford Harbor (Massachusetts), Newark Bay (New Jersey), and Elizabeth River (Norfolk, VA). All three sites have been identified by the Environmental Protection Agency (EPA) as Superfund sites (part of the federal government's program to clean up the nation's uncontrolled hazardous waste sites) and contain high levels of a variety of lipophilic, persistent and toxic contaminants worthy of remediation using Federal funds. All three Superfund sites are highly contaminated with chemical pollutants that are broadly classified as aromatics. New Bedford Harbor is polluted with extremely high levels of polychlorinated biphenyls [37] as well as polychlorinated dibenzo-p-dioxins (PCDD), polychlorinated dibenzofurans (PCD), polycyclic aromatic hydrocarbons (PAH), and several trace metals [37,38]. Newark Bay is most notorious for containing 2,3,7,8-tetrachlorodibenzo-p-dioxin (TCDD) as well as other dioxins [39,40] and also is contaminated with heavy metals, pesticides, PCBs and PAHs [41]. The Elizabeth River is predominantly contaminated with creosote, comprised of a complex mixture of PAHs [42-44].

*F. heteroclitus *from these chronically polluted areas are resistant to the aromatic hydrocarbons in their environment as compared to nearby fish from relatively clean environments [45-52]. Resistance in first and second generation embryos from New Bedford Harbor and Elizabeth River and first generation embryos from depurated Newark Bay fish suggests that differential survival is due to genetic adaptation rather than physiological induction. Investigating and comparing *F. heteroclitus *from these three sites provides the opportunity to study similarities and differences in adaptation to differing chemical pollutant and resistance to general stress conditions among populations.

Previous work to elucidate mechanisms of resistance and the underlying genetic basis in *F. heteroclitus *from these three sites has investigated the refractory phenotype of the xenobiotic metabolizing enzyme cytochrome P4501A (CYP1A) in polluted populations [47,48,53-55], epigenetic silencing through CpG methylation of promoter regions of the CYP1A 5' promoter region [56], and elimination of contaminants through the induction of other phase I, II, and III enzymes [55,57-59], many by way of the aryl hydrocarbon receptor (AHR) pathway (reviewed in [60]). Yet, none of these research efforts has completely accounted for the differences in the resistance phenotypes between polluted and reference site fish in New Bedford Harbor, Newark Bay and Elizabeth River, nor has the genetic basis for resistance been elucidated.

In contrast to a candidate gene approach, our strategy to begin to understand the genetic mechanisms that enable *F. heteroclitus *populations to inhabit these highly polluted sites was to screen the genome for selectively important loci. The premise is that loci under selection will have patterns of variation statistically different from the majority of neutral loci [61]. Loci that have a large difference in allele frequencies between populations with respect to what would be expected under the neutral expectation are outliers. The identification of these outliers provides evidence for which and how many loci may be involved in the evolutionary adaptation to anthropogenic pollution.

Loci can have significantly different frequencies relative to other neutral loci for many reasons. To obviate the detection of outliers due to genetic drift rather than selection, our sampling scheme contrasted each polluted population with two reference populations that were geographically more distant from each other than either was to the polluted population. This provides a control for each Superfund site by identifying which loci are significant outliers relative to two reference sites that are demographically distant from each other. To provide extensive coverage of the genome, we used approximately 300 amplified fragment length polymorphisms (AFLP) [62] to genotype 288 individuals from nine *F. heteroclitus *populations and used a modeling approach to reveal significant outliers. Furthermore, we investigated whether outlier loci were shared among polluted populations, suggesting similar patterns of selection on the genome despite differences in pollutant compositions and local conditions.

## Methods

**Figure 1:**
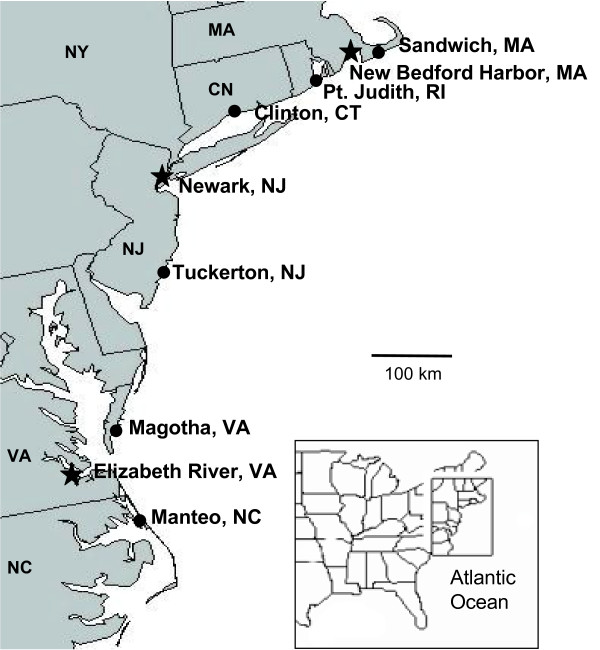
**Sample locations**. Sampling locations for *Fundulus heteroclitus *populations. Circles are reference sites and stars are Superfund sites.

*F. heteroclitus *were collected using minnow traps during the spring of 2005. Fin clip samples from 32 individuals were sampled from each of the nine collection sites along the east coast of the United States (Fig. 1; Table 1). Three of the collection sites were Superfund sites: New Bedford (EPA ID: MAD980731335), Newark (EPA ID: NJD980528996), and Elizabeth River (EPA ID: VAD990710410). Two non-polluted reference sites flanked each Superfund site, approximately equidistant on either side of each polluted site (Fig. 1; Table 1).

**Table 1:** Sample locations

Reference/Superfund	Abbreviation	Geographical location	Latitude (N)	Longitude (W)
Reference	SAND	Sandwich, MA	41°44.0'	70°23.0'
**Superfund**	NBH	New Bedford, MA	41°34.0'	70°54.9'
Reference	PTJ	Point Judith, RI	41°21.7'	71°28.9'
Reference	CLI	Clinton, CT	41°15.3'	72°32.8'
**Superfund**	NEW	Newark, NJ	40°41.2'	74°06.7'
Reference	TUCK	Tuckerton, NJ	39°32.2'	74°19.4'
Reference	MAG	Magotha, VA	37°10.6'	75°56.5'
**Superfund**	ER	Elizabeth River, VA	36°48.5'	76°17.7'
Reference	MAN	Manteo, NC	35°53.8'	75°36.9'

Site locations (Reference or Superfund), sample abbreviations, and geographical locations for *Fundulus heteroclitus *populations.

Genomic DNA was extracted from fin clips using a modified version of Aljanabi and Martinez [63]. Fin clips were incubated at 55°C for two hours in 300 μL of 75 mM NaCl, 25 mM EDTA, and 1% SDS with Proteinase K (3 μL of 20 mg/mL). Following incubation, 0.5 volumes of 7.5 M ammonium acetate were added and DNA was precipitated on ice with the addition of 0.7 volumes of isopropanol. Subsequently, DNA was pelleted through centrifugation and washed with 70% ethanol. DNA was resuspended overnight at 4°C in 0.1× TE.

The AFLP analysis was performed in replicate following the ligation of the DNA for each individual using a modified version of Vos *et al*. [62] to generate approximately 300 loci. Genomic DNA (500 ng) was digested with 5 U EcoRI (New England Biolabs, MA) and 5 U MseI (New England Biolabs, MA) overnight at 37°C in a total volume of 45 μL containing 1× T4 DNA ligase buffer (Epicentre) supplemented with 100 μg/mL BSA. Following incubation, 50 pmol adaptor oligonucleotides (Applied Biosystems) and 1 U T4 DNA ligase (Epicentre) were added and incubated overnight at 16°C. Preselective PCRs were performed in a 15 μL volume using 5 μL of diluted (1:10) ligation product with EcoRI + (C/A) primer (Integrated DNA Technologies; 10 pmol), MseI + (C/A) primer (Integrated DNA Technologies; 10 pmol) and 1 U *Taq*. PCR conditions were 20 cycles of 94°C for 10 sec, 56° for 30 sec, and 72°C for 2 min. Selective Eco + 3NT primers (Integrated DNA Technologies; 10 pmol) labeled with FAM dye at the 5' end and MseI + 3NT primers (Integrated DNA Technologies; 10 pmol) were added to diluted (1:10) pre-selective PCR product in a 15 μL volume. PCR conditions in the first cycle were 94°C for 10 sec, 65°C for 30 sec, and 72°C for 2 min with the annealing temperature reduced by 0.4°C for 12 cycles, then 30 cycles of 94°C for 10 sec, 56°C for 30 sec, and 72°C for 2 min. Semi-automated analysis of the selective PCR products was performed on MegaBACE 1000 DNA sequencing system (GE Healthcare). Peak patterns were calculated using MegaBACE Geneprofiler software v. 1.0 (GE Healthcare). The criteria for distinct peaks were a size between 50 and 400 base pairs and an absolute intensity greater than or equal to 1000. Replicated fragments were obtained from all samples (the same template was used for independent PCRs) and replicate fragments were scored as being present or absent using Peakmatcher software [64]. Peakmatcher software automatically creates marker categories and generates a binary table for the presence and absence of markers based on the minimum 75 percent repeatability of markers across replicates.

### Statistical Analysis

**Figure 2:**
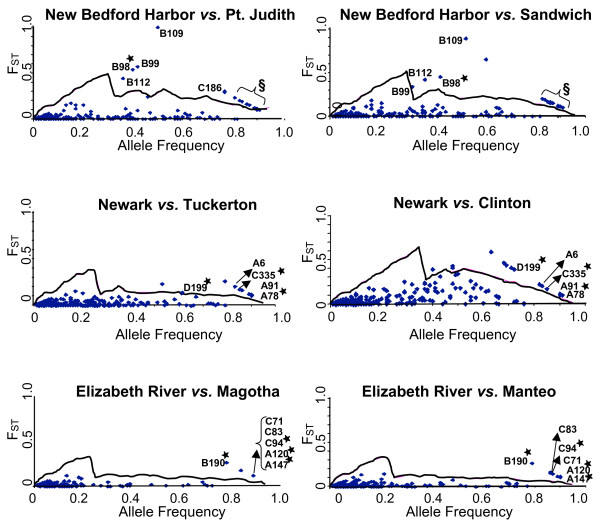
**F_ST _*versus *allele frequency values**. F_ST _values estimated from approximately 300 variable AFLP loci plotted against mean allele frequency. The solid line represents the 0.99 quantile estimated from a simulation model for each comparison. Loci shared among the same Superfund site are labeled with their primer set (letter) and number. Loci shared between Superfund sites are starred. §Shared loci included in these points are: A2, A19, A34, A56, D87, E118, E127, E137, E150, E156, C186, C194, C205, and C252. E118 also is shared between New Bedford Harbor and Elizabeth River populations.

The frequency of band presence allele was calculated using the formula P = 1 - ((N - C)/N)^0.5 ^where N equals the sample size and C is the number of individuals with the band [65]. This formula assumes Hardy-Weinberg equilibrium. However, because AFLPs are dominant markers and heterozygotes are not observed, Hardy-Weinberg equilibrium cannot be directly tested. Due to strong selection or increased mutational rates, some of the loci may not be in Hardy-Weinberg equilibrium. Though not directly comparable, microsatellites are in Hardy-Weinberg equilibrium in these *F. heteroclitus *populations [66]. This calculation also assumes that shared band presence or absence between two individuals is due to common evolutionary origin and not homoplasy. Pairwise F_ST _values between populations were calculated for each locus by the method of Nei [67] with the correction of Nei and Chesser [68] for finite sample sizes, and a null distribution of F_ST _values *versus *allele frequency was simulated using the Winkles program ([69], Fig. 2).

Winkles is based on the model described in Beaumont and Nichols [20] which employs coalescent simulations using the Island model and an infinite alleles mutational model. Samples of the same size and number as the data are simulated, where each sample is taken from a different island. This simulation uses two populations of size *N *diploid individuals, with a set mutation rate, *μ*, and a migration rate, *m*, per generation. Parameters for the simulation are estimated through the calculation F_ST _= 1/(1 + 16*Nm *+ 16*N*μ). The F_ST _value is found by calculating the mean F_ST _from any given pairwise comparison and adjusting that value by -0.0093 to account for the upward bias in the model reported by Wilding et al. [69]; this bias is consistent with previous simulations using Nei's methods to calculate pairwise F_ST _values [70]. The *Nm *factor is calculated by solving for that parameter in the above equation. Each simulation used 10^3 ^and 10^-4 ^as estimates of *N *and μ, respectively. Simulated F_ST _values are relatively unaffected by changing either the sample size of the simulated population or the mutation rate [20]. Five simulations were run on each pairwise comparison to generate an expected null distribution of 25,000 values. Each simulation started with 500 simulation bi-allelic loci in each of the two populations with uniform random distribution and was allowed to drift for 10*N *generations. The 99^th ^percentile of F_ST _values within each of the 40 binned mean allele frequency values (each bin representing a set of 0.025 frequency values from 0 to 1) was calculated after removing monomorphic loci because F_ST _is strongly dependent on allele frequencies [20].

The model we used [20] is robust to a wide range of alternative models such as colonization and stepping-stone [5]. It is likely to detect outliers with unusually high F_ST _values and will identify adaptive selection at one or many loci through pairwise comparisons of populations [5,11]. This model is not able to identify loci under balancing selection and tends to generate discrepancies when numbers of immigrants per generation are unequal, the true population history consists of repeated branching events, or the connectivity of populations is uneven [5]. Isolation, population bottlenecks, and populations which are heterogeneous with respect to their demographic parameters further bias to the model [20]. There is no evidence for isolation and bottleneck history [66] or reduced genetic diversity [71] in our populations. However, if non-homogenous demographic parameters exist (*e.g*., skewed age structure or sex ratios), this model may be biased. Given the relative robustness of the model to identify loci under adaptive selection, we used theoretical *versus *experimentally derived allele frequencies for loci to determine significant deviations from the neutral expectation.

## Results

### Total number of loci among populations

Five different primer combinations (Table 2) were used to amplify approximately 300 loci from 288 individuals from nine different *F. heteroclitus *populations. Among New Bedford Harbor and its reference sites, Sandwich and Point Judith, a total of 296 loci were scored. Of those 296 loci, 11 bands were found to be monomorphic (3.7%). Newark and its two reference sites, Tuckerton and Clinton, had a total of 336 loci, of which 7 loci were monomorphic (2.1%). Elizabeth River and its two reference sites, Magotha and Manteo, had a total of 299 loci, with 4 loci found to be monomorphic (1.3%). Among all populations, 450 distinct loci were scored.

**Table 2:** Primer sequences used in AFLP analyses

Primers	Sequence (5'-3')
***Eco +1***	
*Eco +A*	GACTGCGTACCAATTCA
*Eco +C*	GACTGCGTACCAATTCC
***Mse +1***	
*Mse +A*	GATGAGTCCTGAGTAAA
*Mse +C*	GATGAGTCCTGAGTAAC
***Eco +3***	
*Eco +ACT*	GACTGCGTACCAATTCACT
*Eco +ACC*	GACTGCGTACCAATTCACC
*Eco +AAG*	GACTGCGTACCAATTCAAG
***Mse +3***	
*Mse +AGT*	GATGAGTCCTGAGTAAAGT
*Mse +ATC*	GATGAGTCCTGAGTAAATC
*Mse +CAA*	GATGAGTCCTGAGTAACAA
*Mse +CGA*	GATGAGTCCTGAGTAACGA
	
**Combinations**	
	
A	Eco+ACT and Mse+AGT
B	Eco+ACC and Mse+ATC
C	Eco+AAG and Mse+CAA
D	Eco+ACT and Mse+CGA
E	Eco+ACC and Mse+CAA

### Outlier loci among populations

**Figure 3:**
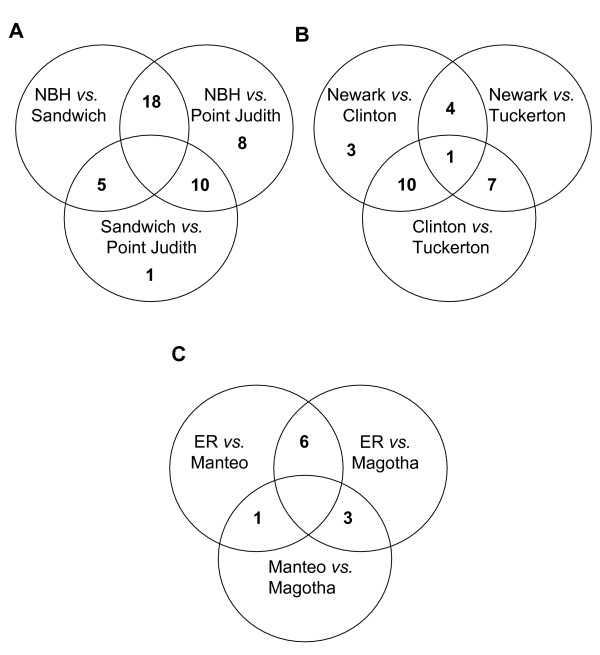
**Venn diagrams of shared outlier loci in each Superfund comparison**. Outlier loci in comparisons of each Superfund populations to both its clean reference sites; numbers in the unions of circles represent outlier loci shared among populations. A) New Bedford Harbor, MA Sandwich, MA and Pt. Judith, RI comparison. B) Newark Bay, NJ, Clinton, CT, and Tuckerton, NJ comparison. C) Elizabeth River, VA, Magotha, VA and Manteo, NC comparison.

**Figure 4:**
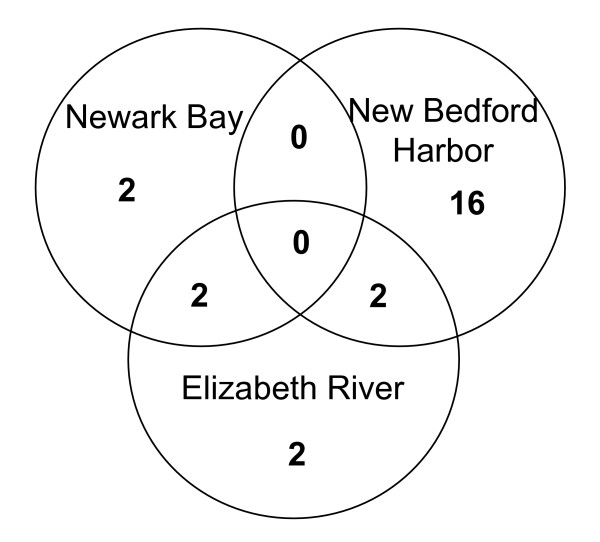
**Venn diagram of shared outlier loci among Superfund populations**. Shared outlier loci among Superfund population comparisons to both clean reference sites; numbers in the unions of circles represent outlier loci shared between two Superfund populations.

In comparisons of the three Superfund sites and their clean reference sites, twenty-four loci show patterns indicative of selection. The criteria for identifying these selective loci are that they were identified as outliers in pairwise comparisons of each Superfund site population relative to its two reference site populations (polluted *versus *both references, analyzed separately, i.e. the union of polluted *versus *reference 1 and polluted *versus *reference 2) but not in comparisons between the reference site populations. Eighteen of these twenty-four loci were found in the New Bedford Harbor comparisons, four were found in the Newark Bay comparisons, and six were found in the Elizabeth River comparisons (Fig. 3). Four of these loci were shared between two Superfund site populations suggesting conserved mechanisms of adaptation (Fig. 4).

In the northern most Superfund site, New Bedford Harbor, 42 loci representing 14% of total analyzed loci were located above the simulated 0.99 quantile in the polluted *versus *one of the references' comparisons. That is, these 42 loci have F_ST _values that lie outside the expected neutral distribution of 99% of all loci. This is more than 10 fold greater than the 3 that are expected by chance from the approximately 300 amplified loci. These 42 loci are outliers in the New Bedford Harbor comparison to the Point Judith, RI reference population (36 loci), the Sandwich, MA reference population (23 loci) or relative to both reference sites (18 loci). The 18 outlier loci found in the comparisons of New Bedford Harbor to both of its reference populations were amplified from three different primer combinations, spanning a 100 base pair range (Fig. 3A). The joint probability (<0.01 squared or <0.0001) indicates that less than one locus should be different in both clean sites *versus *the Superfund site. These 18 loci are thus implicated as separate loci under selection or linked to areas of the genome under selection. There are 16 loci that are outliers when comparing the two reference populations to each other. Only one of these 16 outlier loci is specific to the clean reference sites; the other 15 are also found in the comparison to the New Bedford Harbor Superfund site to one of these reference sites. No locus was an outlier in all pairwise comparisons.

Newark Bay, NJ is close to the phylogeographic boundary that separates northern and southern populations of *F. heteroclitus *[66,72]. The Clinton reference population is on the northern side and the Tuckerton reference population is on the southern side. The Newark Bay Superfund site has 26 outlier loci (8% *versus *1% expected) relative to these two reference sites: 18 (5%) in the comparison with the Clinton reference population and 13 (4%) in the comparison with the Tuckerton reference site population. Four outlier loci are found in both comparisons between the Newark Bay Superfund site and its two clean reference sites (Fig. 3B) and not among clean sites. These four loci are greater than that predicted from the joint probability of differences in both clean sites *versus *the Superfund site. In pairwise comparisons of the two clean reference sites, 18 loci are outliers. Ten of these 18 loci are common outliers between a northern and two different southern populations *i.e*., Clinton and Newark Bay populations and Clinton and Tuckerton populations.

Elizabeth River is the most southern Superfund site. The Elizabeth River population, in comparisons to its two reference site populations, had 9 outlier loci (3%). The Elizabeth River and Magotha reference site comparison had 8 outlier loci (2.7% of the total loci) whereas the Elizabeth River and Manteo reference site comparison had 7 (2.4% of the total loci). Six outlier loci were found in both comparisons (Fig. 3C) and not found in the comparison among clean sites. Among the two reference sites (Magotha and Manteo) only three loci were outliers and none of these were unique to the reference-reference comparison. Two loci were in common with outliers from the Elizabeth River-Magotha comparison and one locus was in common with the Elizabeth River-Manteo comparison.

Among the twenty-three loci that were outliers in comparisons only among Superfund sites and both reference sites, four loci are outliers in two of the three Superfund sites (Fig. 4; Table 3). Two of these four outlier loci are shared between New Bedford Harbor and Elizabeth River populations, and two are shared between Newark Bay and Elizabeth River. None is shared between New Bedford and Newark Bay, nor are any shared among all three Superfund site populations.

**Table 3:** Outlier loci shared among the Superfund site *Fundulus *populations

Population 1 and locus number	Population 2 and locus number	Primer Set
New Bedford Harbor, 19	Elizabeth River, 120	A
New Bedford Harbor, 98	Elizabeth River, 190	B
Newark Bay, 78	Elizabeth River, 147	A
Newark Bay, 335	Elizabeth River, 194	C

**Figure 5:**
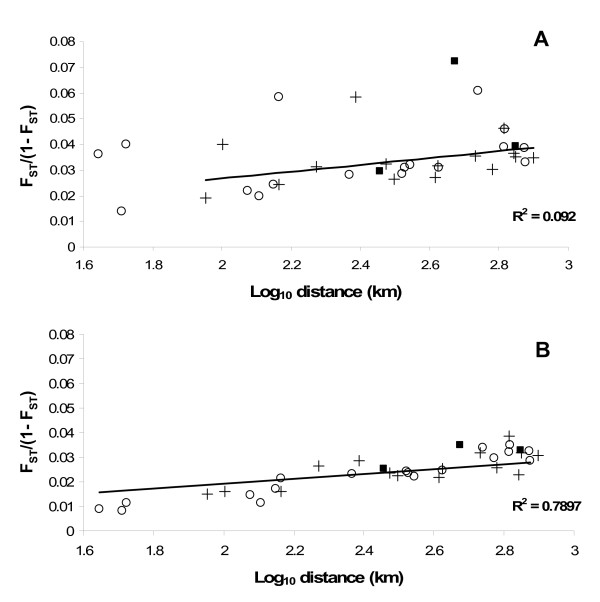
**Geographic *versus *genetic distance**. Relationship between genetic distance and geographic distance. Genetic distance was calculated from the mean F_ST _for each pair of populations with (A) and without (B) outlier loci. Circles represent a pairwise comparison of a Superfund *versus *a reference site, squares represent a Superfund *versus *a Superfund site comparison, and crosses represent a reference *versus *a reference site comparison.

F_ST _values were calculated for comparisons between all sites with and without outlier loci (Table 4). As would be expected, average F_ST _values were higher in all comparisons before the removal of the outliers. The average F_ST _value (with outliers) between New Bedford Harbor and its reference sites is 0.038, between Newark and its reference sites it is 0.039, and between Elizabeth River and its reference sites it is 0.018. Upon removal of the outliers, average F_ST _values fall to 0.010, 0.016, and 0.011 for New Bedford Harbor, Newark Bay, and Elizabeth River, respectively. These values were plotted against log-ten of geographic distance between sites *versus *genetic distance [F_ST_/(1 - F_ST_), [73]]. There is no apparent pattern in the distribution of pairwise comparisons corresponding to reference-reference, polluted-reference, or polluted-polluted sites. When outliers were included in the calculation of average F_ST _and plotted against distance, there was no significant linear relationship (R^2 ^= 0.092, p = .0721). Upon removal of the outliers, there was a significant and strong linear relationship (R^2 ^= 0.79, p < 0.0001) between geographic and genetic distance (Fig. 5). Mantel tests that account for multiple comparisons confirmed the significance of both relationships (data not shown). This relationship indicates that 79% of the variability in the neutral genetic distance (without outlier loci) between sites can be explained by geographic distance.

**Table 4:** Pairwise F_ST _values with and without outlier loci

	SAND	NBH	PTJ	CLI	NEW	TUCK	MAG	ER	MAN
SAND		0.0090	0.0157	0.0258	0.0238	0.0213	0.0220	0.0315	0.0298

NBH	0.0361		0.0112	0.0168	0.0249	0.0299	0.0318	0.0318	0.0277

PTJ	0.0399	0.0399		0.0149	0.0228	0.0219	0.0250	0.0338	0.0308

CLI	0.0313	0.0245	0.0190		0.0211	0.0278	0.0309	0.0288	0.0370

NEW	0.0286	0.0294	0.0283	0.0584		0.0112	0.0239	0.0338	0.0328

TUCK	0.0270	0.0309	0.0264	0.0584	0.0197		0.0230	0.0218	0.0247

MAG	0.0365	0.0388	0.0303	0.0355	0.0310	0.0322		0.008	0.0159

ER	0.0387	0.0393	0.0460	0.0294	0.0722	0.0320	0.0140		0.0144

MAN	0.0349	0.0330	0.0350	0.0464	0.0607	0.0318	0.0243	0.0217	

Mean F_ST _between populations of *Fundulus heteroclitus *with and without outlier loci. Below diagonal: mean F_ST _including outlier loci. Above diagonal: mean F_ST _without outlier loci. Average of F_ST _values below diagonal is 0.034 and 0.023 after the removal of outlier loci.

## Discussion

Multiple *F. heteroclitus *populations have independently evolved adaptive resistance to complex suites of pollutants [45-52,74,75]. These different populations provide independent contrasts for identifying loci involved in adaptation. We identified loci suggestive of adaptation for each polluted population by identifying outlier loci in the polluted population relative to two nearby reference populations. These loci are outliers because they are statistically different from the neutral distribution among populations. Only loci exhibiting a non-neutral distribution in comparisons of the polluted population *versus *both a north and south reference population were considered to be adaptive. Through this comparison, we are more likely to identify loci whose non-neutral distribution is due to pollution rather than geography. Similarly, while the model used to identify outlier loci has a false positive rate of approximately 7% [11], it is unlikely that the same loci will be falsely identified in multiple comparisons (*i.e*., in the polluted population *versus *both a north and south reference populations). In each of the Superfund sites, 1% to 6% (four to 18 loci out of approximately 300) of amplified fragments were identified as being loci under selection or linked to areas of the genome under selection. Four of these loci were outliers in two separate Superfund population comparisons.

We only consider loci exhibiting a non-neutral distribution in comparisons of the polluted population *versus *both a north and south reference population to be adaptive. These populations make up a geographic triangle formed among the northern and southern clean reference populations and a latitudinally intermediate polluted population (Fig. 1). This double comparison ensures that we are not identifying loci that differ simply due to genetic drift or clinal variation common to this species. This contrast, in addition to the joint comparison among populations, address most of the possible neutral or demographic models. Population isolation can alter allele frequencies among populations. One would expect that a single population that suffered from unique isolation would have significantly greater F_ST _values among many loci in comparison to similarly geographically distance populations that were not uniquely isolated. This demographic explanation does not fit the data for two reasons: 1) it is the statistically different F_ST _value for a few loci in comparison to all other loci that we define as being important, and 2) all non-outlier loci follow the more common demographic trend of isolation by distance (Fig. 5). However, differences in F_ST _values also can result if loci under functional constraints evolve more slowly than loci without functional constraints. Thus, loci with large F_ST _values would have few, if any constraints, relative to the hundreds of other AFLP loci. However, our comparisons were based on both a significant F_ST _between both reference sites *versus *a polluted site **and **insignificant differences among reference sites (as well as a difference from the permutation model, see methods). Because we are using three criteria (significant difference *versus *the joint distribution in two reference sites, lack of a difference among reference sites, and a statistical difference from a neutral permutation model), it seems most parsimonious to suggest that these outlier loci are due to natural selection. However, lack of Hardy-Weinberg equilibrium or recent mutations also might cause loci to be outliers. We suggest that the most obvious cause for this evolved difference is chronic exposure to the aromatic hydrocarbons and other anthropogenic pollutants; yet, we cannot explicitly control every variable in natural environments. Other selective forces also could be different between the three sites. For instance, site complexity differs among the nine sites with the three polluted sites tending to be less complex (have less edges) than the reference sites. Thus, predation or food availability might differ among sites. Similarly, salinity might affect food availability or absorption, and although all populations inhabit brackish waters, the Elizabeth River population is less coastal than the reference populations to which it is compared. Under controlled laboratory conditions, survival differs among fish from clean populations exposed to polluted sediments and fish from polluted populations exposed to clean sediments. This phenomenon points towards adaptation to anthropogenic contaminants rather than differing local conditions for the differences seen between polluted and reference populations. Thus we postulate that outlier loci are due to pollution, especially those loci shared among separate Superfund populations.

Most of the outlier loci are unique to a single polluted population rather than shared across polluted populations (Fig. 4). One explanation for the lack of shared loci is that different loci are involved in the adaptation to a particular pollutant or stress. Alternatively, some of these outliers might be linked to the same locus in the different populations and only appear to be different because the locus under selection dragged different polymorphisms to fixation. This could occur because different polymorphisms existed in the different ancestral populations.

Resistance to pollution is a modern phenotype in *F. heteroclitus *due to recent exposure (approximately within the last 60 years), suggesting that *F. heteroclitus *have rapid evolutionary responses with respect to their environment. Our data and other data on survival and development indicate that populations of *Fundulus *have adapted to local pollutants and thus selection has favored a few alleles. Resistance phenotypes resulting from rapid evolution have been well documented in plants [76] and benthic invertebrates [77] in response to metals as well as in insects in response to pesticides [78] and depend both on population dynamics as well as the strength of selection. *F. heteroclitus *populations residing in chronically polluted areas provide an advantageous situation whereby strong selective pressures and rapid evolution can be studied. *F. heteroclitus *have high standing genetic variation [79], high reproductive potential [80], limited home ranges [81] and large population sizes exceeding 10,000 in a single tidal creek [66]. These attributes can and have resulted in locally adapted *F. heteroclitus *populations. Adaptation due to positive selection often reduces genetic variation among natural populations because of selective sweeps. For example, reduced genetic variation has occurred in brown rats resistant to the rodenticide, warfarin [25,82,83], tobacco budworm exposed to the pyrethroid insecticide [84], and the human malarial parasite, *Plasmodium falciparum*, exposed to antimalarial agents [85]. However, genetic diversity is not reduced in the polluted *F. heteroclitus *populations compared to the reference site populations for either neutral markers [71,86,87] or gene expression [88]. Maintenance of genetic diversity in these populations subjected to significant selection most likely represents steady influx of alternative alleles by migration. If migration and resulting gene flow is strong enough to prevent the reduction of genetic diversity at non-selected loci, it suggests that selection at adaptively important loci is equally strong. Importantly, with constant influx of allelic variation at loci without adaptive value, there should be fewer spurious allelic differences among populations. Thus, shared loci between Superfund populations are likely to be affected by selection and therefore biologically important.

Among three *F. heteroclitus *populations inhabiting highly polluted Superfund sites and flanking reference populations, 63 different loci (14% of the collective 450 loci) have F_ST _values outside the 99% quantile. Using all loci (*i.e*., including outliers) our F_ST _values based on AFLP (0.038, 0.039, and 0.018 for New Bedford Harbor, Newark Bay and Elizabeth River, respectively) are approximately one-half of those found for microsatellites (0.077, 0.068, and 0.043, respectively [66]) although these genetic measures are difficult to compare due to differences in genomic coverage and mutation rates [89]. Using AFLPs, McMillan *et al*. [71] found similar F_ST _values for the New Bedford Harbor population (0.056). For the Elizabeth River population, Mulvey *et al*. [86] also found similar F_ST _values (0.014) using allozymes. Notice that these calculated F_ST _values use all loci and do not distinguish between neutral and non-neutral loci. If selection affects the frequency of alleles among these molecular markers, the perceived genetic distance (F_ST_) will be exaggerated.

The neutral hypothesis is a powerful tool to explore differences among populations [90]. However, in order to test evolutionary hypotheses, one needs to distinguish between neutral and non-neutral loci. Among populations for each Superfund site, the genetic distances among local populations are affected by the outlier loci. New Bedford Harbor and Newark Bay populations are more differentiated in comparison to their reference site populations than the Elizabeth River populations (F_ST _values of 0.038 and 0.039 *versus *0.018) because the Elizabeth River population has the fewest outlier loci (2.4% – 2.7%) in comparison to neutral loci. These differences among Superfund sites do not exist upon removal of outliers: F_ST _values among loci without outlier values are similar for New Bedford Harbor, Newark Bay and Elizabeth River (0.01, 0.016, and 0.011, respectively). With outliers, there is no relationship between F_ST _values and geographic distance. However, upon removal of outlier loci, there is a strong relationship between genetic and geographical distance indicating an equilibrium model of isolation-by-distance. Similar findings have been shown in other *F. heteroclitus *studies [66,87], with the intertidal snail [69], and sea trout [91]. Not surprisingly, these data indicate that loci with unusually large F_ST _values have a large and potentially misleading effect on the perceived genetic distance among populations. The 63 outliers exhibit this effect; once removed from the data set, the neutral expectation of increasing genetic distance with geographic distance holds true. For twenty-four of these outlier loci, this non-neutral distribution is most likely caused by evolution by natural selection due to pollution or another strong selective force unique to the polluted sites since the geographical effect was taken into account through the comparison of the polluted sites with both a north and south reference population. Ten other loci have a larger than expected distance at the north-south phylogenetic boundary and likely reflect the historic split among northern and southern *F. heteroclitus *populations [92-94]. Outlier loci in reference-reference pairwise comparisons likely reflect genetic drift although some may be due to selection. While we can only speculate why these and the remaining 29 loci affect the relationship between genetic and geographic distance, this illustrates the need to distinguish among potentially selected and neutral loci to determine expected differences and posit hypotheses.

## Conclusion

Contrasting populations that experience different selective pressures provides insight into evolution by natural selection. Our goal is to understand the genetic basis of adaptive resistance to pollution in chronically contaminated natural populations. Future analyses will address whether polymorphisms between populations are functional and potentially responsible for conferring resistance in populations adapted to chronic exposure to chemical pollutants in the different Superfund sites. We have shown that between 1 to 6% of loci are implicated as being under selection or linked to areas of the genome under selection in three distinct *F. heteroclitus *populations that reside in polluted Superfund estuaries. Shared loci affected by natural selection among polluted sites indicate that there may be a similar mechanism of resistance in these different populations. This study suggests that multiple loci may be involved in adaptation and a few of these loci have a generalized adaptive response.

## Authors' contributions

LMW designed experiments, carried out laboratory and statistical analysis, and drafted the manuscript. MFO designed experiments, assisted on statistical analysis, and helped draft the manuscript.
